# Physical seed dormancy in pea is genetically separable from seed coat thickness and roughness

**DOI:** 10.3389/fpls.2024.1359226

**Published:** 2024-02-27

**Authors:** Owen R. Williams, Jacqueline K. Vander Schoor, Jakob B. Butler, Valérie F. G. Hecht, James L. Weller

**Affiliations:** ^1^ School of Natural Sciences, University of Tasmania, Hobart, TAS, Australia; ^2^ ARC Centre of Excellence for Plant Success in Nature and Agriculture, University of Tasmania, Hobart, TAS, Australia

**Keywords:** *Pisum*, seed dormancy, seed coat, domestication, permeability, gritty, flavonoid

## Abstract

**Introduction:**

The seeds of wild pea (*Pisum*) exhibit marked physical dormancy due to impermeability of the seed coat to water, and the loss of this dormancy is thought to have been critical for domestication. Wild pea seed coats are also notably thick and rough, traits that have also reduced during domestication and are anecdotally linked to increased permeability. However, how these traits specifically interact with permeability is unclear.

**Methods:**

To investigate this, we examined the genetic control of differences in seed coat characteristics between wild *P. sativum* ssp. *humile* and a non-dormant domesticated *P. s. sativum* accession in a recombinant inbred population. QTL effects were confirmed and their locations refined in segregating F_4/5_ populations.

**Results:**

In this population we found a moderate correlation between testa thickness and permeability, and identified loci that affect them independently, suggesting no close functional association. However, the major loci affecting both testa thickness and permeability collocated closely with Mendel’s pigmentation locus A, suggesting flavonoid compounds under its control might contribute significantly to both traits. We also show that seed coat roughness is oligogenic in this population, with the major locus independent of both testa thickness and permeability, suggesting selection for smooth seed was unlikely to be due to effects on either of these traits.

**Discussion:**

Results indicate loss of seed coat dormancy during domestication was not primarily driven by reduced testa thickness or smooth seededness. The close association between major permeability and thickness QTL and Mendel’s 'A' warrant further study, particularly regarding the role of flavonoids.

## 1 Introduction

Pea (*Pisum sativum*) is considered to be one of the world’s earliest-domesticated crops. Its divergence from ancestral wild forms is estimated to have occurred over 10,000 years ago in the Fertile Crescent, broadly in parallel with several other legume and cereal crops (Lev-Yadun et al., 2000; Zohary et al., 2012). Two critical steps in its domestication were the loss of pod dehiscence and seed dormancy (Ladizinsky, 1985, 1987), changes which were likely to have improved the efficiency returns for early farmers (Abbo et al., 2011).

Similar changes have occurred during the domestication of other legume crops, and the first robust insights into their genetic and molecular control has recently begun to emerge from work in species such as soybean and common bean. Genes influencing pod dehiscence (“shattering”) in these species affect pod lignification, and variously encode NAC and MYB transcription factors and a dirigent-like protein (Dong et al., 2014; Funatsuki et al., 2014; Di Vittori et al., 2021; Parker et al., 2021). In soybean, physical seed dormancy is determined by a major locus *Hs-1* which has been equivocally associated with variation in distinct genes influencing either polysaccharide or calcium content of the seed coat (Jang et al., 2015; Sun et al., 2015). More recently, a single major locus that governs seed coat permeability in the Andean genepool of common bean has also been implicated in control of polysaccharide content (Soltani et al., 2021).

In pea, reduced pod dehiscence in domesticated material has been primarily attributed to the major locus *DEHISCENT PODS* (*DPO*) (Blixt, 1972; Bordat et al., 2011) which has yet to be identified at the molecular level. The genetic basis for reduction in seed dormancy is even less well understood. In wild peas physical seed dormancy is imposed by a thick, hard seed coat which may prevent water entry for many months, whereas seed coats of domesticated lines are much thinner and readily permeable to water (Smýkal et al., 2014). In addition, detailed anatomical and biochemical characterizations have revealed the upper section of macrosclereid cells (light line) to be a major barrier to water uptake in dormant seeds (Janská et al., 2019), and lower proanthocyanidin levels and less extensive cell wall deposition in seed coats of non-dormant accessions (Hradilová et al., 2017, 2019). However, systematic genetic analysis of these traits and their relationship to permeability has not been undertaken.

In addition to thickness and permeability, another seed coat trait anecdotally linked to dormancy in pea is roughness, characterized as a granular “gritty” surface texture that reflects a regular pattern of size variation within the outer layer of macrosclereid cells. This feature is absent in domesticated pea but ubiquitous in wild germplasm, and varying degrees of testa roughness are also characteristic of (or more prominent in) the wild forms of several other legumes, including lentil, sweet pea and chickpea (Lersten and Gunn, 1982; Sedláková et al., 2021). In pea, this trait has been reported as a monogenic trait under the control of the *GRITTY* (*GTY*) locus (Marx, 1969) and in view of its restriction to wild material, it is inferred to have been strongly selected against early in pea domestication.

The aim of this study was to examine the functional basis of seed physical dormancy in pea, by defining the genetic control and relationships of seed coat traits, including thickness, permeability and roughness in a wild x domesticated RIL population previously analyzed for flowering time (Williams et al., 2022).

## 2 Materials and methods

### 2.1 Plant material and growing conditions

An F_8_+ recombinant inbred line (RIL) population (*n*=137) was derived by single seed descent from the F_2_ of a cross between the wild *P. sativum* ssp. *elatius* line JI1794 (a representative of the northern “humile” subgroup; Hellwig et al., 2022) and the cultivar NGB5839. This population was described previously described by Weller et al. (2012) and Williams et al. (2022). This population was grown under long-day (LD) conditions (16 hours light – 8 hours dark), with 4 replicate plants per genotype. Plants were grown in a 1:1 gravel:vermiculite mixture, topped with sterilized potting mix which included controlled release fertilizer. Seeds were harvested after plants had completely senesced and dried, and were stored for at least a month to ensure they had fully matured prior to their use in permeability analyses.

### 2.2 Phenotypic evaluation

**Figure 1:**
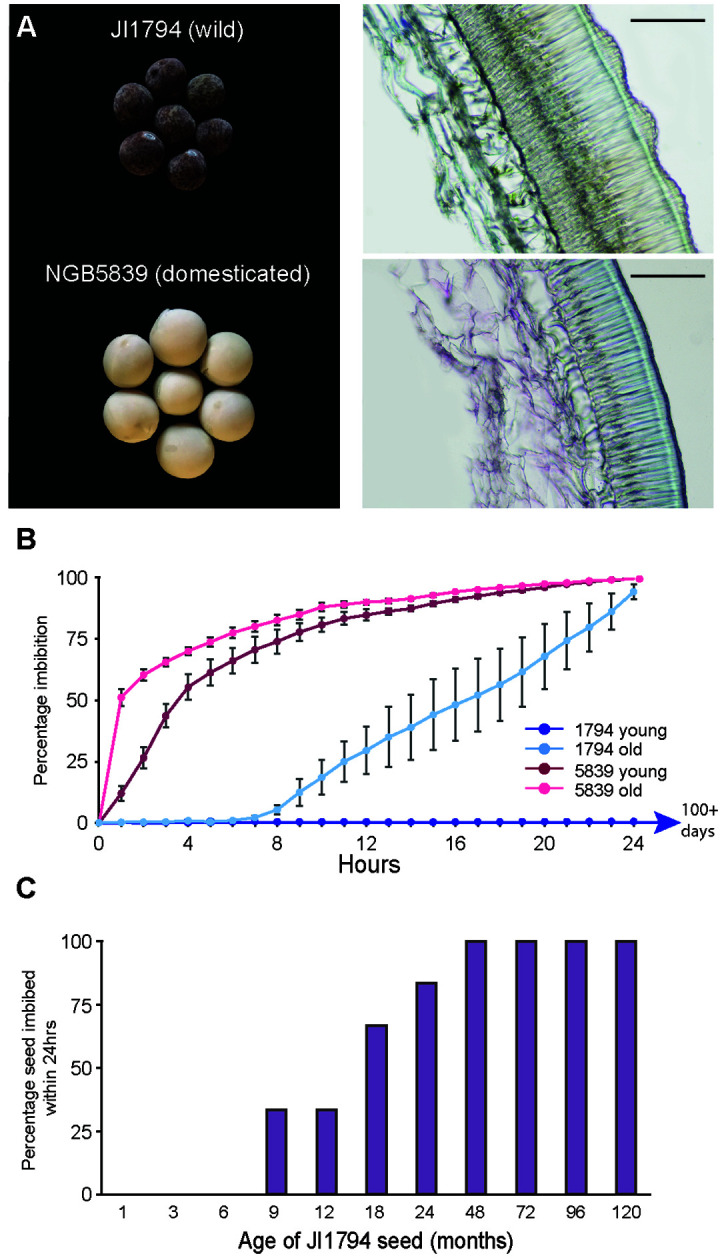
Comparison of seed coat traits in wild and domesticated pea. **(A)** Wild *P. s. humile* line JI1794 seed and testa section with visible roughness and domesticated *P. s. sativum* cultivar NGB5839 seed and testa section. Scale bars = 0.1 mm. **(B)** Time to fully imbibe in water for young (1 month old mature dried) and old (2 year old mature dried) seed from both wild and domesticated pea. Wild young seed had not imbibed within 100 days, after which point the experiment was concluded. **(C)** Effect of seed age on the ability of JI1794 seed to imbibe within 24h. All seed was mature and dry.

The harvested seeds of the RIL population and parental lines were assessed for several traits potentially related to physical dormancy (**Table 1**). To estimate seed coat permeability, we measured time to fully imbibe with water by submerging seed in water and retrieving, drying and weighing at regular intervals until there was no further increase in mass due to water uptake. This was measured in both young (1 month old mature dried) and old (2 year old mature dried) seed. The relative water uptake capacity of the dry seed was characterized by the relative increase in weight of the seeds at full imbibition. The thickness of the testa was initially measured on detached fragments using a micrometer, but to verify the precision of these measures, further seed coat dimensions were determined from transverse sections mounted on slides, from three representative seeds for each genotype. Images were captured at 10X and 40X magnification to allow for accurate delineation of cell layers and anatomical zones. The total thickness of the testa and the width of distinct component layers (**Supplementary Figure S1**) were measured using the line tool in ImageJ (Schneider et al., 2012), while roughness was measured as the ratio of the outer cuticle length over the light line length (**Figure 1A**, **Supplementary Figure S1**). Mean seed weight was recorded from ten representative seeds per genotype. Variation in seed pigmentation traits was also recorded, including for Mendel’s *A* locus governing flower and seed anthocyanin content and several other classical seed pigmentation loci.

**Table 1:** Traits phenotyped in the JI1794 x NGB5839 RIL population.

Trait	Abbreviation	Description	Loci reference
Seed permeability	PERM	Time for seed to fully imbibe after being submerged in water	
Testa thickness (micrometer)	TT_mm	Measurement of testa thickness *via* micrometer	
Testa thickness (sectioning)	TT	Measurement of testa thickness from sectioning	
Testa section thickness	TT-A to TT-F	As above, but component sections of the testa (**Supplementary Figure 1**)	
Seed coat roughness	GRIT (*GTY*)	Measured as ratio of the outer cuticle length over the light line length	(Marx, 1969)
Seed weight	SW	Mean dry weight of ten representative seeds	
Seed water uptake capacity	WUP	Relative weight gain after fully imbibed with water	
Green testa	O, GLA	Presence of green testa colour	(Lamprecht, 1959) (Blixt, 1962)
Black spot	Fs	Presence of black spots on testa	(Lamprecht, 1961)
Black hilum	Pl	Presence of black hilum colour	(Lamprecht, 1948) (Balarynová et al., 2022)
Marbling	M	Presence of marbled testa patterning	(Lamprecht, 1948)
Flower pigmentation	A	Presence of flower pigmentation	(Hellens et al., 2010)

### 2.3 Genotyping and QTL analysis

Genomic DNA was extracted from young leaflets of each genotype using the CTAB extraction protocol (Doyle and Doyle, 1987), and DarTseq markers (Sansaloni et al., 2011) generated using Diversity Array Technology Pty. Ltd. (Canberra, Australia). A total of 4,599 markers (4,575 DArT and 24 gene-based markers) were used to construct a linkage map (previously described by Williams et al., 2022). The linkage map was subsequently reduced to 3073 markers for quantitative trait loci (QTL) analysis by removing every third marker, which improved computational efficiency while having minimal effect on mapping resolution, and some linkage groups inverted to maximize synteny with the *Pisum* genome assembly (Kreplak et al., 2019).

QTL analysis was performed using MapQTL v6 (Van Ooijen, 2009), following Williams et al. (2022). In brief, QTLs were defined by a > 3 LOD score and identified using the interval mapping (IM) function. Iterative searches for additional QTLs were performed using the restricted Multiple QTL Model (rMQM) function, which increases the power of QTL analysis by reducing residual variances attributed to previously identified QTLs (cofactors). The amount of variation explained by each QTL was estimated using the coefficient of determination (R^2^) which is represented as the Phenotypic Variance Explained (PVE).

### 2.4 Advanced generation segregating populations and fine mapping

QTL regions of interest were refined in segregating F_3_, F_4_ and F_5_ progeny derived from specific individuals in the original F_2_ population. Where possible, these progenies were selected to be homozygous (fixed) for other relevant loci influencing the focus trait. Mapping resolution within these selected QTL regions was increased through use of additional high-resolution melt (HRM) markers, either already available or newly developed from pea transcript sequences with selection guided by DArT marker positions in the RIL linkage map or the high-density consensus map of Tayeh et al. (2015). Progenies of these advanced generations were grown under the conditions described above, and phenotyped for permeability, roughness (as a binary presence/absence trait) and testa thickness (*via* micrometer).

## 3 Results

### 3.1 Characterisation of seed dormancy in representative wild and domesticated lines

A comparison between the wild *P. s. humile* line JI1794 and the domesticated *P. s. sativum* cultivar NGB5839 illustrates the significantly thicker testa in the wild line, and the undulating outer surface characteristic of the ‘gritty’ phenotype (**Figure 1A**). Recently-matured (one-month-old; “young”) dry seed of JI1794 did not imbibe or germinate even after more than 100 days of immersion in water, whereas similar seed of NGB5839 started to take up water within one hour of immersion, reached 50% imbibition within 4h (**Supplementary Figure S2**) and reached 100% imbibition after 24h (**Figure 1B**). To examine the potential influence of seed age on imbibition, we also compared JI1794 seed of various ages post-harvest. Unlike young seed, 80% of older seed that had been stored for two years in a relatively stable, cool and low-humidity indoor environment commenced imbibition within 6h of immersion and reached the fully-imbibed state by 24h (**Figure 1B**, failure to imbibe not shown). In contrast, this same period of storage had no effect on imbibition of NGB5839. A comparison of storage duration on permeability in JI794 (in this case detecting partial imbibition after 24h of immersion) revealed that the initial complete impermeability persisted for between 6 and 9 months, after which imbibition increased to reach 100% after 4 years of storage at relatively constant temperature and humidity (**Figure 1C**). This suggests that the impermeability of wild seed coats is lost as the seed ages.

### 3.2 Variation in seed dormancy-related traits in the RIL F_8_ population

We next examined variation for seed coat traits potentially related to dormancy and domestication in the JI1794 x NGB5839 RIL population. This included the thickness of the seed coat and its component layers, permeability (time to fully imbibe) and the roughness or ‘grittiness’ of the seed coat, as the main traits potentially related to dormancy. We also examined seed weight, water uptake capacity and various pigmentation features. Mean data for all traits and a correlation matrix are provided in **Supplementary Table S1** and **Supplementary Figure S3**, with notable correlations discussed below.

**Figure 2:**
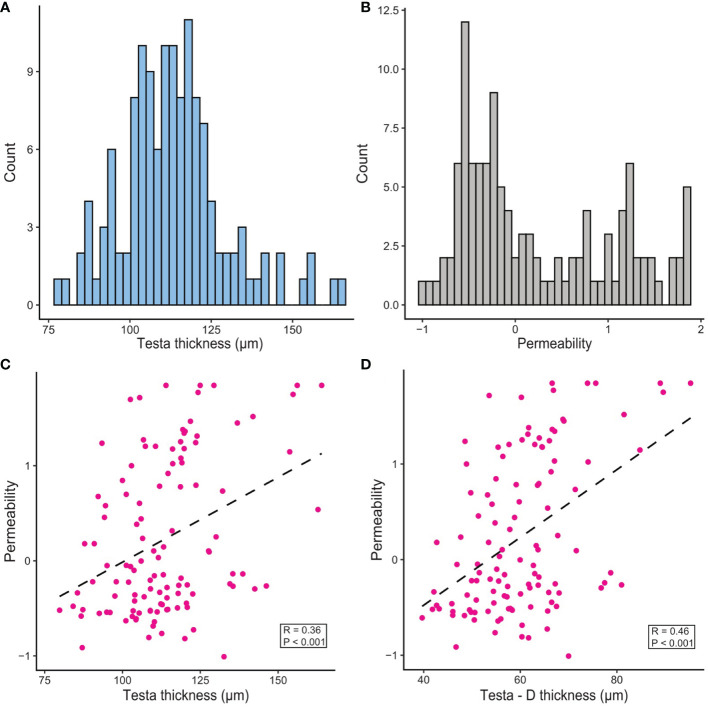
Distribution and correlation of dormancy-related phenotypes in the RIL F_2_ population. **(A)** Testa thickness. **(B)** Permeability (measured as log_10_[time to fully imbibe in days]). **(C)** Correlation of testa thickness with permeability. **(D)** Correlation of testa section D thickness (see **Supplementary Figure 1**) with permeability.

Total testa thickness varied from 76 – 160 µm and exhibited a near-normal distribution in the population (**Figure 2A**). To examine whether specific anatomically distinct layers within the testa might vary in thickness and how this might contribute to the total thickness, we also defined specific zones within the transverse section (testa sections TT-A through TT-F; **Supplementary Figure S1**) and measured these individually. The largest component was section TT-D of the macrosclereid cell layer, which is the main component of the testa. This section contributed 38 – 64% of the total variation for testa thickness across the population. Testa thickness showed a positive correlation with water uptake capacity (r = 0.20, p < 0.05), along with a positive correlation with seed coat roughness (r = 0.28, p < 0.01). Testa thickness also showed a moderate negative correlation with seed weight (r = -0.26, p < 0.01), but interestingly a much stronger negative correlation with seed weight was found for the testa sections TT-A, TT-B and TT-C (r = -0.54 - -0.77, p < 0.001). Likewise, the presence of flower pigmentation had a moderately positive correlation with testa thickness (r = 0.30, p < 0.001), but this correlation was much higher when considering the thickness of testa section D (r = 0.49, p < 0.001).

Permeability varied widely, with imbibition time ranging from 2 hours to more than 70 days, heavily skewed towards a shorter time to imbibe. To adjust this skew prior to analysis, permeability scores were log transformed, creating a bimodal distribution (**Figure 2B**). This measure of permeability was moderately correlated to total testa thickness, with thicker testa leading to a longer time to imbibe (r = 0.34, p < 0.001, **Figure 2C**). Again, this effect was more pronounced when considering the thickness of testa section D (r = 0.41, p < 0.001, **Figure 2D**). Permeability was not affected by seed coat roughness (r = 0.01, p > 0.05). Likewise, no correlation was detected between permeability and water uptake capacity (r = -0.04, p > 0.05), suggesting that seed water capacity was unrelated to imbibition rate. Time to fully imbibe was also positively correlated with presence of flower pigmentation (r = 0.55, p < 0.001).

### 3.3 QTL analysis of seed dormancy-related traits

**Figure 3:**
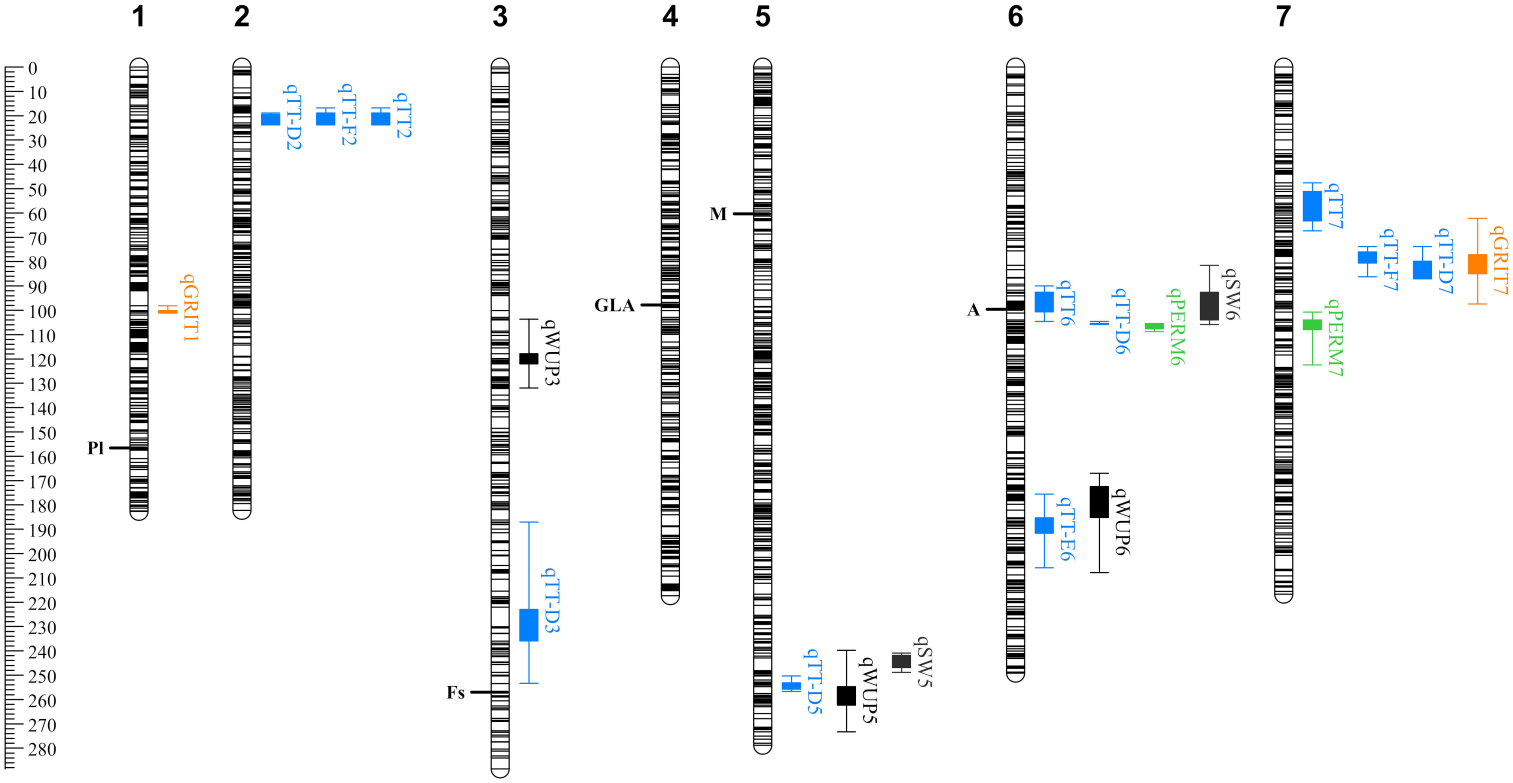
QTL discovered for seed physical characteristics and dormancy-related traits in the *Pisum sativum* RIL F_8_ population. Scale is in cM. QTL nomenclature follows **Tables 1** and **2**. Box and whiskers represent 1-LOD and 2-LOD intervals, respectively, around each QTL peak. Colors indicate broad trait categories, with permeability in green, seed coat roughness (*GRITTY*) in orange, testa thickness in blue and other physical characteristics in black. Previously discovered pigmentation loci (**Table 1**) are indicated at the position of their QTL peaks in this analysis.

Variability in seed permeability to water was found to be under genetic control in this population, with two QTLs discovered on chromosomes 6 (q*PERM6*) and 7 (q*PERM7*), respectively explaining 33.0% and 9.1% of the observed variation (**Table 2**). The q*PERM6* locus was located over the region containing Mendel’s *A* (**Figure 3**), raising the possibility that *A* itself might influence permeability *via* its effects on seed-coat composition. In contrast *qPERM7* was in a region distinct from any known loci likely to have an influence on seed coat properties.

**Table 2:** QTLs discovered for seed dormancy related traits in the cross.

Trait	QTL	Chr/LG	Linkage map position (cM)	Genome position (bp)	PVE (%)	LOD	Peak marker
Seed permeability	qPERM6	Chr6/II	106.578	77,687,086	33.0	11.22	3563452_1
	qPERM7	Chr7/VII	107.014	223,271,526	9.1	3.63	3542137_3
Seed coat roughness	qGRIT1	Chr1/VI	100.48	240,747,079	44.5	18.48	3565980_1
	qGRIT7	Chr7/VII	82.654	167,195,112	8.8	4.74	3641990_4
Testa-D thickness	qTT-D6	Chr6/II	105.403	83,667,261	33.3	19.03	4655870_3
	qTT-D2	Chr2/I	21.039	10,511,954	19.0	12.35	5252181_1
	qTT-D7	Chr7/VII	83.971	172,063,975	10.6	7.51	3554532_3
	qTT-D5	Chr5/III	254.671	551,212,215	10.2	7.27	3545539_2
	qTT-D3	Chr3/V	230.078	355,504,338*	5.0	3.78	4662852_2
Testa-E thickness	qTT-E6	Chr6/II	189.699	367,810,783	10.1	3.07	3546899_3
Testa-F thickness	qTT-F2	Chr2/I	22.039	10,511,954	30.6	12.18	5252181_1
	qTT-F7	Chr7/VII	77.61	162,499,192	9.6	4.35	3563336_3
Testa thickness (total)	qTT2	Chr2/I	21.039	10,511,954	25.5	11.01	5252181_1
	qTT6	Chr6/II	96.845	64,232,569	13.7	6.40	3537228_3
	qTT7	Chr7/VII	52.994	98,865,458	9.5	4.59	4663696_3
Seed water uptake capacity	qWUP5	Chr5/III	258.937	557,511,547	12.7	4.62	4660931_2
	qWUP3	Chr3/V	120.456	159,233,156	10.4	3.85	3564112_4
	qWUP6	Chr6/II	176.179	333,875,501	7.9	3.00	4662859_3
Seed weight	qSW5	Chr5/III	244.887	530,128,486	35.1	13.08	3566597_1
	qSW6	Chr6/II	98.906	67,581,138*	10.3	4.50	3553850_3

*The BLAST location of peak marker sequence in the Pisum sativum genome assembly was to a different chromosome than expected based on linkage group synteny, so position of next closest marker reported.

Three QTL were detected for total testa thickness (*TT*) as measured by sectioning, on chromosomes 2, 6 and 7. Of these, the *qTT2* locus explained 25.5% of the observed variation and was located near the top of chromosome 2. *qTT7* was located in a region distinct from *qPERM7*, but *qTT6* was closely collocated with *qPERM6*. Collectively, these comparisons indicate that the genetic control of physical dormancy is at least in part independent of testa thickness, but also imply the possibility of a relationship between these traits. Additional QTL were detected when testa thickness was measured by micrometer, but the presence of collocated QTL for other traits at these additional loci suggests a measurement precision issue (see below).

In order to examine whether variation for testa thickness might reflect a specific contribution from certain cell layers or structural features we also looked at the genetic control of the thickness of the six distinct testa layers described above (**Supplementary Figure S1**). Significant loci were found only for testa layers D, E and F, with five QTL for *qTT-D2* explaining 78.1% of the variation. The three strongest *qTT-D* QTL collocated with the *qTT2*, *qTT6* and *qTT7* loci, and explained 53% of the variation. Two weaker *TT-D* loci were detected on chromosomes 5 and 3, together explaining another 15% of the variation. The only locus detected for layer E was in a position on chromosome 6 distinct from that of *qTT6* and *qTT-D6*, while the two loci detected for layer F co-located with the *qTT2/qTT-D2* and *qTT7/qTT-D7* loci. Overall, these results indicated that the observed variation in total testa thickness was primarily due to variation in layer D, and revealed the contribution of three minor loci on chromosomes 3, 5 and 6.

In addition to Mendel’s *A*, the four seed pigmentation traits measured all related to well-known classical loci, and major QTL were detected in the expected genomic locations when analyzed as qualitative traits (**Figure 3**, shown as peak position). These included the hilum pigmentation locus *Pl* on chromosome 1 (Lamprecht, 1948; Balarynová et al., 2022), the seed-coat “marbling” locus *M* on chromosome 5 (Lamprecht, 1948; Ellis et al., 2023), the seed coat speckling locus *Fs* on chromosome 3 (Lamprecht, 1961; Ellis et al., 2023) and the seed coat ground color locus *GLA* on chromosome 4 (Lamprecht, 1959; Blixt, 1962). Each of these also had a secondary QTL which collocated with Mendel’s *A*, reflecting their likely dependence on the action of *A*. However, with the exception of the A locus itself, none of these were collocated with effects on testa thickness or permeability.

Seed coat roughness has long been noted as a key distinguishing feature of wild and domesticated pea seeds; a difference attributed to a major Mendelian locus *GRITTY* located on chromosome 1. Assessment of this trait has typically been made somewhat subjectively based on the degree of friction encountered when rubbing seeds together (Ellis et al., 2023). We quantified it more objectively, examining seed coat sections under the light microscope and taking quantitative measurements of the surface undulations. A major QTL explaining 45% of the variation (*qGRIT1*) was detected on chromosome 1, in the expected position of the *GTY* locus. A second, minor locus (*qGRIT7*) explaining a further 8.8%, was located near the cluster of *TT* loci on chromosome 7. These results suggest that the *GRITTY* trait has no major genetic association with testa permeability, and only a minor association with testa thickness. Interestingly, an additional QTL for testa thickness was discovered tightly linked to *qGRIT1* when measuring testa thickness using a micrometer. Given this QTL was not present when using the more accurate sectioning measurement, this likely reflects the additional apparent thickness conferred by the undulations of the seed coat when roughness is present, highlighting the need for precision in measurements when working at this scale.

Genetic control of seed water uptake and seed weight was also detected in this cross, with QTL for these traits often collocated with testa thickness QTL (**Figure 3**). A common locus for both was found on chromosome 5, with an additional seed weight QTL present in the QTL cluster on chromosome 6, and an additional water uptake QTL collocating with the testa thickness section E QTL on chromosome 6 (in a distinct location). There was also an independent QTL for water uptake on chromosome 3, suggesting additional physiological phenomena beyond testa thickness affecting this trait.

### 3.4 Validation of permeability and roughness QTL in advanced generations

As the largest permeability QTL was located on chromosome 6 in a region also featuring Mendel’s *A* locus and QTL for physical seed characteristics, we attempted to refine the position and determine whether it might be distinct from the *A* gene. We also further refined the position of the main seed roughness QTL (*GRITTY*) as a next step in status as a key domestication trait.

**Figure 4:**
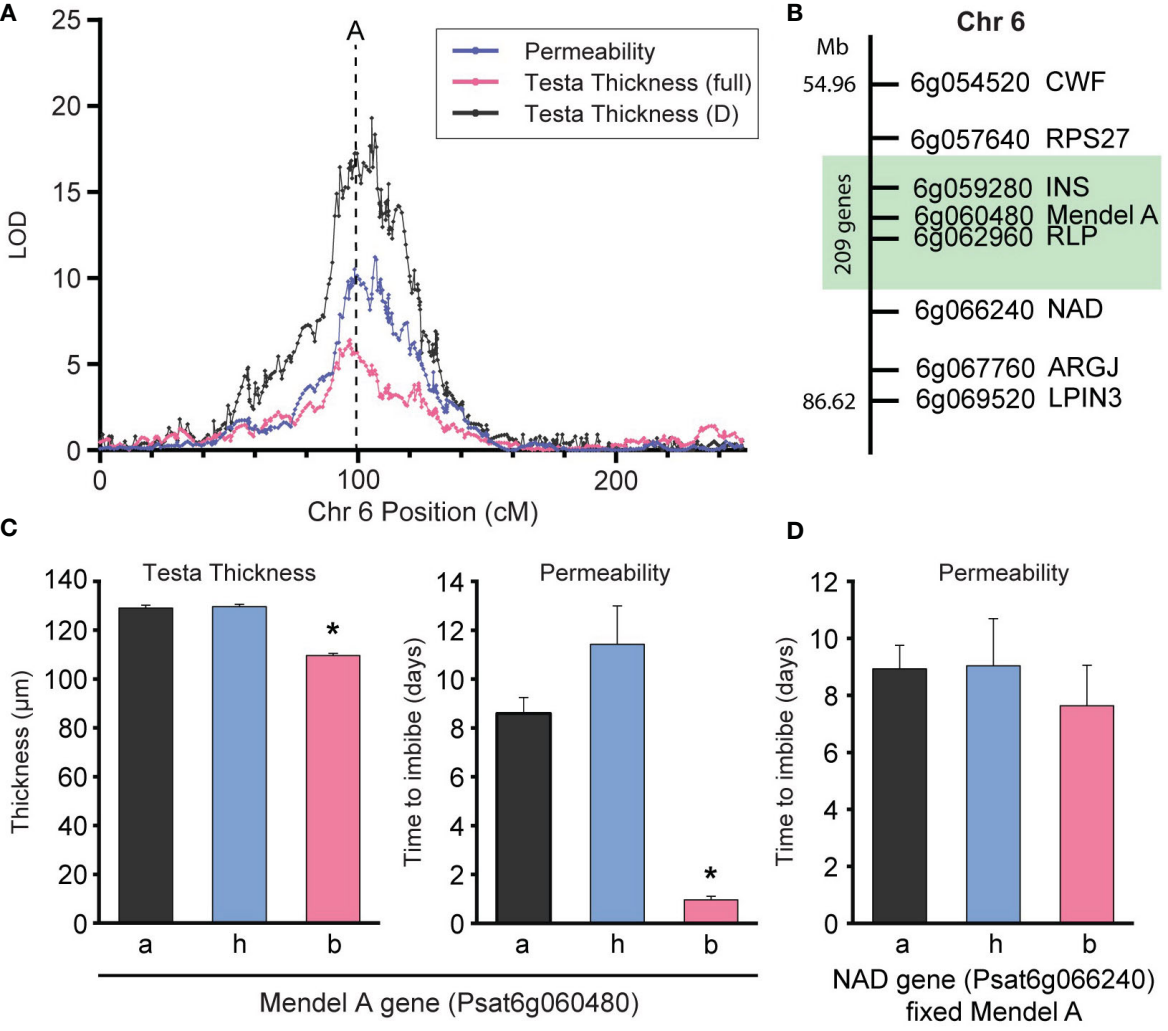
QTL6 impacts on testa thickness and permeability in advanced populations. **(A)** LOD profile of permeability, testa thickness (full) and testa section D thickness QTL near the Mendel’s *A* locus, which is marked with a dashed line. **(B)** Fine mapping of the Mendel’s *A* region on chromosome 6, showing the region (green) where there is significant effect of the genes on permeability and testa thickness **(C)** Effect of genotype at the Mendel’s *A* locus on testa thickness and permeability in an advanced F_4_ & F_5_ populations respectively. The * indicates significant differences between genotypes a/h and b (P < 0.01). **(D)** Effect of genotype of the NAD gene on permeability in advanced F_5_ population when the region of Mendel’s *A* is fixed. Data in C & D populations were fixed for the wild allele at RPS27 and CWF. Full segregation data is available in **Supplementary Figure S4**.

The presence of the wild allele at QTL6 conferred significantly increased testa thickness (t_129_ = -3.49, p < 0.001) and lower permeability (t_112_ = -6.75, p < 0.001) compared to the domesticated, conforming to findings in the RIL population for this QTL (**Figure 4A**). To validate this QTL, further advanced segregation populations were developed using single seed descent from an F_2_ line which was segregating for the *qPERM6* locus (markers *LF* and *A*) and fixed at all other loci of interest (specifically fixed for the domesticated allele at *qPERM7*), thereby allowing the analysis of this QTL as a near Mendelian trait in the F_4_ and F_5_ populations developed. For greater resolution around *qPERM6* region (spanning between 38.4cM and the A_1 marker) eight additional markers (**Supplementary Table S2**) were genotyped across these populations (**Figure 4B**). Markers were ordered based on recombinant frequency or (if there was no recombination) by order in the *Medicago* (v4.0, Tang et al., 2014) or *P. sativum* (v1a, Kreplak et al., 2019) reference genomes.

Results showed all individuals in these populations were fixed for wild alleles between RPS27 and CWF and segregating at all other loci. Phenotyping these populations (separately, due to the destructive nature of the phenotyping) confirmed the segregation of testa thickness in the F_4_ and permeability in the F_5_ (**Figure 4C**). Homozygosity for the domesticated allele at the Mendel’s *A* gene resulted in significantly thinner testa (t_47_ = 13.1, p < 0.001) and higher permeability (t_47_ = 3.4, p = 0.001) compared to the heterozygous and homozygous wild individuals, indicating RPS27 as the upper boundary for this QTL effect. Further, when only individuals fixed for wild Mendel’s *A* were analyzed, genotype at NAD had no significant effect on permeability in the F_5_ (**Figure 4D**), indicating NAD as the lower boundary for this QTL effect.

**Figure 5:**
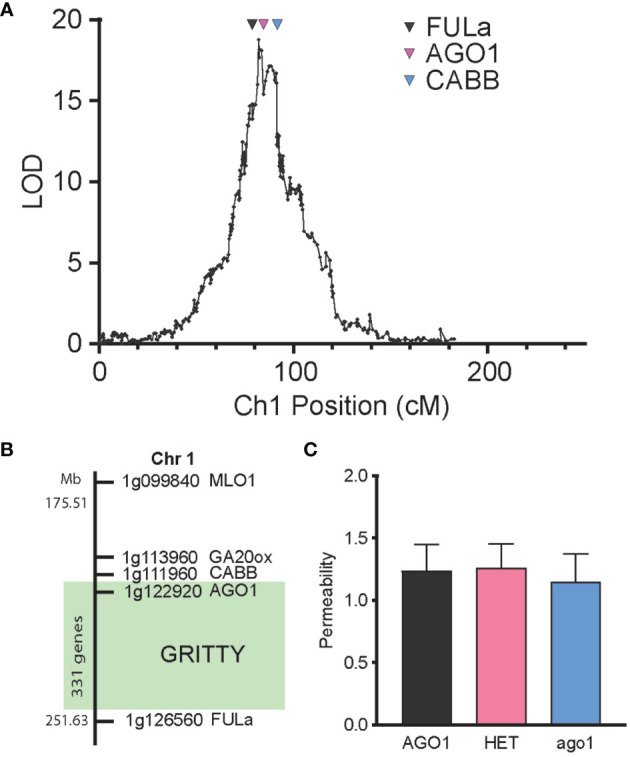
Genetic separation of seed coat roughness from permeability. **(A)** LOD profile of seed coat roughness QTL on chromosome 1 and the position of three markers FULa, AGO1 and CABB. **(B)** Mapping of the *GRITTY* region, showing the position of key markers on chromosome 1 and the region (in green) within which *GRITTY* has been mapped. **(C)** Effect of genotype at the AGO1 locus on permeability in an advanced F_4_ population.

Of the additional markers previously genotyped in the RIL population (Williams et al., 2022), the markers FULa, AGO1 and CABB were found to span the peak of the seed coat roughness QTL (**Figure 5A**). To confirm the validity of this QTL, advanced F_4_ populations (and a subsequent F_5_ population) were generated by single seed descent from lines in the F_2_ cross which were segregating for the region between CABB and FULa on chromosome 1 but fixed for the wild allele at *qPERM6* and the domesticated allele at *qPERM7*. Phenotyping these populations showed segregation of seed coat roughness, with the QTL effect able to be localized between FULa and CABB, a region that spans 37 MB and contains 331 genes (**Figure 5B**, **Supplementary Table S3**). Notably, genotype at AGO1 had no effect on seed permeability (**Figure 5C**), confirming that seed coat roughness had no detectable impact on the permeability of the seed coat to water.

## 4 Discussion

Loss of physical seed dormancy is widely considered to be one of the key domestication-related modifications in pea (Ladizinsky, 1987; Zohary, 1989; Abbo et al., 2011), and primarily involves an increase in permeability of the seed coat (Smýkal et al., 2014). However, little is known about the genetic or functional basis for this change. It has clearly been accompanied by a reduction in testa thickness (**Figure 1**, Plitmann and Kislev, 1989; Hradilová et al., 2017), an association that has raised the question of a possible relationship between these traits. Our genetic analysis of dormancy-related seed traits in a previously-generated wild x domesticated RIL population has shown only moderate correlation between testa thickness and permeability (**Figure 2**), and identified loci that affect them independently (**Figure 3**), suggesting there is no close functional association.

One major factor shaping the results of this study is the segregation of Mendel’s *A* gene in the population. This gene encodes the bHLH component of the well-known MYB/WD40/bHLH (MWB) complex which has a central role in regulation of flavonoid pathways (Xu et al., 2015; Wen et al., 2020), and naturally-arising *a* mutations result in complete loss of anthocyanin and derivatives in seed coats, stems and flowers (Hellens et al., 2010). A reduction in anthocyanins and proanthocyanidins in seed coats has been associated with increased permeability and reduced thickness in several species including pea (Werker et al., 1979; Lepiniec et al., 2006; Smýkal et al., 2014; Sedláková et al., 2023) and consistent with this, we found QTL for testa thickness and permeability closely co-located with *A* (**Figure 3**). The most straight-forward explanation of this co-location is that both *qTT6* and *qPERM6* represent direct effects of *A* itself, with compounds under its control contributing significantly to both traits. A possible direct effect of *A* on permeability could also be examined through isolation of induced *a* mutants, or through more detailed genetic dissection of the region.

However, despite this plausible functional link, loss of *A* function is unlikely to have been an early and critical step in reduction of physical dormancy given that functional *A* alleles are widespread in the domesticated pea germplasm (Hellens et al., 2010; Holdsworth et al., 2017). Apart from *qPERM6*, we only detected only one other permeability locus, *qPERM7*, with a much weaker effect (9% PVE, vs. 33% for *qPERM6*). In principle, this locus could have contributed to the reduction in dormancy during domestication, but it is somewhat surprising that it was the only other locus detected and explains so little of the variance. This result could imply that transition to increased permeability early in domestication was incremental and strongly polygenic, with *qPERM7* representing the strongest effect, and a further substantial increase due to *qPERM6* arose only later with the appearance of the *a* mutation.

An alternative interpretation is that, despite the proximity of *qPERM6* to *A*, and the precedent for the function of *A* orthologs in testa permeability, the *qPERM6* effect may not be solely or primarily due to *A*, but reflects variation at another gene in the QTL interval that arose earlier than the *a* mutation. If this were the case, a similar analysis with a landrace or other primitive accession carrying functional *A* alleles as the domesticated parent would be expected to reveal an effect at *qPERM6* independent of *A*. One such study found no evidence for a dormancy QTL in the *A* region in a wild x domesticated (JI1794 x Slow) population monomorphic for *A* (Weeden, 2007). However this study employed small populations (n≈50) and low marker density, and warrants re-examination.

Genetic control of physical seed dormancy has been characterized to some extent in several grain legumes. In some species, 4-6 QTL have been detected (Isemura et al., 2010, 2012; Kongjaimun et al., 2012), while in other species such as lentil, bean and soybean, the trait has been attributed to single major QTL (Ladizinsky, 1985; Sun et al., 2015; Soltani et al., 2021). To date only one testa permeability locus has been characterized at the molecular level. In soybean, permeability of the intact seed coat in domesticated germplasm depends on a single major-effect locus (*Hs-1*) modified by additional quantitative variation (Sun et al., 2015). *Hs-1* encodes a calcineurin-like protein expressed in the macrosclereid layer, and increased permeability of domesticated forms has been functionally associated with a specific amino acid substitution (Sun et al., 2015). This study also found no evidence for an effect of *Hs-1* on testa thickness. Two other genes critical for physical dormancy have been identified by reverse genetics in the model legume *Medicago truncatula*, where mutations in class II KNOX gene *KNOX4* and the keto-acyl synthase *KCS12* reduce long-chain fatty acid content in the cuticular layer of the seed coat and increase its permeability (Chai et al., 2016, 2021). A mungbean ortholog of *KNOX4* was also found to contribute to seed dormancy in a wild x cultivated *Vigna radiata* cross (Laosatit et al., 2022). Other genes potentially linked to physical dormancy in legumes include a truncated phospholipid sterol acyltransferase *VsPAT1* in *V. stipulacea* (Takahashi et al., 2023), and a truncated tandem duplicate of a pectin acetylesterase *PAE-8-2* in bean (Soltani et al., 2021). However, map locations of their pea orthologs on chromosomes 3, 4 and 5 indicate none of these genes are a candidate for either of the pea *qPERM* loci detected in this study. Similarly in pea, 14 differentially expressed candidate genes for dormancy were found between wild and cultivated *P. elatius* (Hradilová et al., 2017), but none were located within the confidence intervals of the present loci.

Loss of seed coat roughness is a characteristic feature of the domesticated *Pisum* germplasm and its strong early selection in parallel with thinness and permeability has historically suggested some association with one or both of these traits. Most earlier studies have assessed roughness somewhat subjectively and as a Mendelian trait. As expected, our more detailed quantitative analysis did identify a major QTL explaining 45% of the variation in the location of the classical *GTY* locus, but this was not co-located with QTL for testa thickness or permeability. This implies that the selection for the recessive *gty* allele during domestication is unlikely to be due to effects on either of these traits.

One general caveat of our study is that our assay for permeability used seeds stored in a stable environment and then tested at a constant temperature. This clearly did not capture the complex daily and seasonal environmental fluctuations likely to be experienced by a dormant seed in a natural environment. We therefore cannot definitively exclude a role for *GTY* and have likely not detected the full range of adaptive variation for seed dormancy represented by the parental lines. Nevertheless, the systematic analysis presented here does prepare the way for future more detailed studies and a comparison with related species.

## Data availability statement

The original contributions presented in the study are included in the article/**Supplementary Material**. Further inquiries can be directed to the corresponding authors.

## Author contributions

OW: Writing – original draft, Writing – review & editing, Data curation, Formal Analysis, Investigation, Methodology. JS: Data curation, Formal Analysis, Investigation, Methodology, Writing – original draft, Writing – review & editing. JB: Data curation, Formal Analysis, Writing – original draft, Writing – review & editing. VH: Formal Analysis, Investigation, Methodology, Supervision, Writing – original draft, Writing – review & editing. JW: Conceptualization, Data curation, Funding acquisition, Investigation, Methodology, Project administration, Supervision, Writing – original draft, Writing – review & editing.

## References

[B1] Abbo S. Rachamim E. Zehavi Y. Zezak I. Lev-Yadun S. Gopher A. (2011). Experimental growing of wild pea in Israel and its bearing on Near Eastern plant domestication. Ann. Bot. 107, 1399–1404. doi: 10.1093/aob/mcr081 21527420 PMC3101147

[B2] Balarynová J. Klčová B. Sekaninová J. Kobrlová L. Cechová M. Z. Krejčí P.. (2022). The loss of polyphenol oxidase function is associated with hilum pigmentation and has been selected during pea domestication. New Phytol. 235, 1807–1821. doi: 10.1111/nph.18256 35585778

[B3] Blixt S. (1962). Studies in induced mutations in peas. Acta Hort Genet. Landskrona 20, 95–110.

[B4] Blixt S. (1972). Mutation genetics in Pisum. Agri Hortique Genetica 30, 1–293.

[B5] Bordat A. Savois V. Nicolas M. Salse J. Chauveau A. Bourgeois M.. (2011). Translational genomics in legumes allowed placing in silico 5460 unigenes on the pea functional map and identified candidate genes in *Pisum sativum* L. G3: Genes| Genomes| Genet. 1, 93–103. doi: 10.1534/g3.111.000349 PMC3276132 22384322

[B6] Chai M. Queralta Castillo I. Sonntag A. Wang S. Zhao Z. Liu W.. (2021). A seed coat-specific β-ketoacyl-CoA synthase, KCS12, is critical for preserving seed physical dormancy. Plant Physiol. 186, 1606–1615. doi: 10.1093/plphys/kiab152 33779764 PMC8260136

[B7] Chai M. Zhou C. Molina I. Fu C. Nakashima J. Li G.. (2016). A class II KNOX gene, KNOX4, controls seed physical dormancy. Proc. Natl. Acad. Sci. 113, 6997–7002. doi: 10.1073/pnas.1601256113 27274062 PMC4922145

[B8] Di Vittori V. Bitocchi E. Rodriguez M. Alseekh S. Bellucci E. Nanni L.. (2021). Pod indehiscence in common bean is associated with the fine regulation of PvMYB26. J. Exp. Bot. 72, 1617–1633. doi: 10.1093/jxb/eraa553 33247939 PMC7921299

[B9] Dong Y. Yang X. Liu J. Wang B.-H. Liu B.-L. Wang Y.-Z. (2014). Pod shattering resistance associated with domestication is mediated by a NAC gene in soybean. Nat. Commun. 5, 3352. doi: 10.1038/ncomms4352 24549030

[B10] Doyle J. Doyle J. (1987). A rapid DNA isolation procedure for small quantities of fresh leaf tissue. Phytochemical Bulletin 19, 11–15.

[B11] Ellis N. Hofer J. Sizer-Coverdale E. Lloyd D. Aubert G. Kreplak J.. (2023). Recombinant inbred lines derived from wide crosses in Pisum. Sci. Rep. 13, 20408. doi: 10.1038/s41598-023-47329-9 37990072 PMC10663473

[B12] Funatsuki H. Suzuki M. Hirose A. Inaba H. Yamada T. Hajika M.. (2014). Molecular basis of a shattering resistance boosting global dissemination of soybean. Proc. Natl. Acad. Sci. 111, 17797–17802. doi: 10.1073/pnas.1417282111 25468966 PMC4273335

[B13] Hellens R. P. Moreau C. Lin-Wang K. Schwinn K. E. Thomson S. J. Fiers M. W.. (2010). Identification of Mendel's white flower character. PloS One 5, e13230. doi: 10.1371/journal.pone.0013230 20949001 PMC2952588

[B14] Hellwig T. Abbo S. Ophir R. (2022). Phylogeny and disparate selection signatures suggest two genetically independent domestication events in pea (*Pisum* L.). Plant J. 110, 419–439. doi: 10.1111/tpj.15678 35061306 PMC9303476

[B15] Holdsworth W. L. Gazave E. Cheng P. Myers J. R. Gore M. A. Coyne C. J.. (2017). A community resource for exploring and utilizing genetic diversity in the USDA pea single plant plus collection. Hortic Res. 4, 17017. doi: 10.1038/hortres.2017.17 28503311 PMC5405346

[B16] Hradilová I. Duchoslav M. Brus J. Pechanec V. Hýbl M. Kopecký P.. (2019). Variation in wild pea (*Pisum sativum* subsp. *elatius*) seed dormancy and its relationship to the environment and seed coat traits. PeerJ 7, e6263. doi: 10.7717/peerj.6263 30656074 PMC6336014

[B17] Hradilová I. Trněný O. Valkova M. Cechová M. Janská A. Prokešová L.. (2017). A combined comparative transcriptomic, metabolomic, and anatomical analyses of two key domestication traits: pod dehiscence and seed dormancy in pea (*Pisum* sp.). Front. Plant Sci. 8, 542. doi: 10.3389/fpls.2017.00542 28487704 PMC5404241

[B18] Isemura T. Kaga A. Tabata S. Somta P. Srinives P. Shimizu T.. (2012). Construction of a genetic linkage map and genetic analysis of domestication related traits in mungbean (*Vigna radiata*). PloS One 7, e41304. doi: 10.1371/journal.pone.0041304 22876284 PMC3410902

[B19] Isemura T. Kaga A. Tomooka N. Shimizu T. Vaughan D. A. (2010). The genetics of domestication of rice bean, *Vigna umbellata* . Ann. Bot. 106, 927–944. doi: 10.1093/aob/mcq188 20880934 PMC2990660

[B20] Jang S.-J. Sato M. Sato K. Jitsuyama Y. Fujino K. Mori H.. (2015). A single-nucleotide polymorphism in an endo-1, 4-β-glucanase gene controls seed coat permeability in soybean. PloS One 10, e0128527. doi: 10.1371/journal.pone.0128527 26039079 PMC4454576

[B21] Janská A. Pecková E. Sczepaniak B. Smýkal P. Soukup A. (2019). The role of the testa during the establishment of physical dormancy in the pea seed. Ann. Bot. 123, 815–829. doi: 10.1093/aob/mcy213 30534972 PMC6526324

[B22] Kongjaimun A. Kaga A. Tomooka N. Somta P. Vaughan D. A. Srinives P. (2012). The genetics of domestication of yardlong bean, Vigna unguiculata (L.) Walp. ssp. *unguiculata* cv.-gr. *sesquipedalis* . Ann. Bot. 109, 1185–1200. doi: 10.1093/aob/mcs048 22419763 PMC3336956

[B23] Kreplak J. Madoui M.-A. Cápal P. Novák P. Labadie K. Aubert G.. (2019). A reference genome for pea provides insight into legume genome evolution. Nat. Genet. 51, 1411–1422. doi: 10.1038/s41588-019-0480-1 31477930

[B24] Ladizinsky G. (1985). The genetics of hard seed coat in the genus *Lens* . Euphytica 34, 539–543. doi: 10.1007/BF00022952

[B25] Ladizinsky G. (1987). Pulse domestication before cultivation. Econ Bot. 41, 60–65. doi: 10.1007/BF02859349

[B26] Lamprecht H. (1948). The variation of linkage and the course of crossing over. Agri Hortique Genetica 6, 10–48. doi: 10.1371/journal.pcbi.1002462

[B27] Lamprecht H. (1959). The inheritance of colors of a seeds of *Pisum* . Agri Hort Genet. Landskrona 17, 1–18.

[B28] Lamprecht H. (1961). Die Genenkarte von *Pisum* beinormaler Struktur der Chromosomen. Agri Hortique Genetica 19, 360–401.

[B29] Laosatit K. Amkul K. Yimram T. Chen J. Lin Y. Yuan X.. (2022). A class II KNOX gene, *KNAT7-1*, regulates physical seed dormancy in mungbean [*Vigna radiata* (L.) Wilczek]. Front. Plant Sci. 13. doi: 10.3389/fpls.2022.852373 PMC8965505 35371162

[B30] Lepiniec L. Debeaujon I. Routaboul J.-M. Baudry A. Pourcel L. Nesi N.. (2006). Genetics and biochemistry of seed flavonoids. Annu. Rev. Plant Biol. 57, 405–430. doi: 10.1146/annurev.arplant.57.032905.105252 16669768

[B31] Lersten N. Gunn C. (1982). Testa characters in tribe Vicieae, with notes about tribes Abreae, Cicereae, and Trifolieae (Fabaceae) (Washington, DC: US Dept. of Agriculture, Agricultural Research Service).

[B32] Lev-Yadun S. Gopher A. Abbo S. (2000). The cradle of agriculture. Science 288, 1602–1603. doi: 10.1126/science.288.5471.1602 10858140

[B33] Marx G. (1969). A new seed gene. Pea News Letter 1, 11–12.

[B34] Parker T. A. Lo S. Gepts P. (2021). Pod shattering in grain legumes: emerging genetic and environment-related patterns. Plant Cell 33, 179–199. doi: 10.1093/plcell/koaa025 33793864 PMC8136915

[B35] Plitmann U. Kislev M. (1989). “Reproductive changes induced by domestication,” in Advances in Legume Biology. Eds. Stirton C. Zarucchi J. (St. Louis: Missouri Botanical Garden Press), 487–504.

[B36] Sansaloni C. Petroli C. Jaccoud D. Carling J. Detering F. Grattapaglia D.. (2011). Diversity Arrays Technology (DArT) and next-generation sequencing combined: genome-wide, high throughput, highly informative genotyping for molecular breeding of *Eucalyptus* . BMC Proc. 5, 1–2. doi: 10.1186/1753-6561-5-S7-P54

[B37] Schneider C. A. Rasband W. S. Eliceiri K. W. (2012). NIH Image to ImageJ: 25 years of image analysis. Nat. Methods 9, 671–675. doi: 10.1038/nmeth.2089 22930834 PMC5554542

[B38] Sedláková V. Hanáček P. Grulichová M. Zablatzká L. Smýkal P. (2021). Evaluation of seed dormancy, one of the key domestication traits in chickpea. Agronomy 11, 2292. doi: 10.3390/agronomy11112292

[B39] Sedláková V. Zeljković S. Ć. Štefelová N. Smýkal P. Hanáček P. (2023). Phenylpropanoid content of chickpea seed coats in relation to seed dormancy. Plants 12, 2687. doi: 10.3390/plants12142687 37514301 PMC10384132

[B40] Smýkal P. Vernoud V. Blair M. W. Soukup A. Thompson R. D. (2014). The role of the testa during development and in establishment of dormancy of the legume seed. Front. Plant Sci. 5, 351. doi: 10.3389/fpls.2014.00351 25101104 PMC4102250

[B41] Soltani A. Walter K. A. Wiersma A. T. Santiago J. P. Quiqley M. Chitwood D.. (2021). The genetics and physiology of seed dormancy, a crucial trait in common bean domestication. BMC Plant Biol. 21, 1–17. doi: 10.1186/s12870-021-02837-6 33482732 PMC7821524

[B42] Sun L. Miao Z. Cai C. Zhang D. Zhao M. Wu Y.. (2015). GmHs1-1, encoding a calcineurin-like protein, controls hard-seededness in soybean. Nat. Genet. 47, 939–943. doi: 10.1038/ng.3339 26098868

[B43] Takahashi Y. Sakai H. Ariga H. Teramoto S. Shimada T. L. Eun H.. (2023). Domesticating *Vigna stipulacea*: chromosome-level genome assembly reveals *VsPSAT1* as a candidate gene decreasing hard-seededness. Front. Plant Sci. 14. doi: 10.3389/fpls.2023.1119625 PMC10149957 37139108

[B44] Tang H. Krishnakumar V. Bidwell S. Rosen B. Chan A. Zhou S.. (2014). An improved genome release (version Mt4. 0) for the model legume Medicago truncatula. BMC Genomics 15, 1–14. doi: 10.1186/1471-2164-15-312 24767513 PMC4234490

[B45] Tayeh N. Aluome C. Falque M. Jacquin F. Klein A. Chauveau A.. (2015). Development of two major resources for pea genomics: the GenoPea 13.2K SNP Array and a high-density, high-resolution consensus genetic map. Plant J. 84, 1257–1273. doi: 10.1111/tpj.13070 26590015

[B46] Van Ooijen J. (2009). MapQTL 6, Software for the mapping of quantitative trait loci in experimental populations of diploid species (Wageningen, Netherlands: Kyazma BV).

[B47] Weeden N. F. (2007). Genetic changes accompanying the domestication of *Pisum sativum*: is there a common genetic basis to the ‘domestication syndrome’ for legumes? Ann. Bot. 100, 1017–1025. doi: 10.1093/aob/mcm122 17660515 PMC2759201

[B48] Weller J. L. Liew L. C. Hecht V. F. Rajandran V. Laurie R. E. Ridge S.. (2012). A conserved molecular basis for photoperiod adaptation in two temperate legumes. Proc. Natl. Acad. Sci. 109, 21158–21163. doi: 10.1073/pnas.1207943110 23213200 PMC3529011

[B49] Wen W. Alseekh S. Fernie A. R. (2020). Conservation and diversification of flavonoid metabolism in the plant kingdom. Curr. Opin. Plant Biol. 55, 100–108. doi: 10.1016/j.pbi.2020.04.004 32422532

[B50] Werker E. Marbach I. Mayer A. (1979). Relation between the anatomy of the testa, water permeability and the presence of phenolics in the genus *Pisum* . Ann. Bot. 43, 765–771. doi: 10.1093/oxfordjournals.aob.a085691

[B51] Williams O. Vander Schoor J. K. Butler J. B. Ridge S. Sussmilch F. C. Hecht V. F.. (2022). The genetic architecture of flowering time changes in pea from wild to crop. J. Exp. Bot. 73, 3978–3990. doi: 10.1093/jxb/erac132 35383838 PMC9238443

[B52] Xu W. Dubos C. Lepiniec L. (2015). Transcriptional control of flavonoid biosynthesis by MYB–bHLH–WDR complexes. Trends Plant Sci. 20, 176–185. doi: 10.1016/j.tplants.2014.12.001 25577424

[B53] Zohary D. (1989). Pulse domestication and cereal domestication: How different are they? Econ Bot. 43, 31–34. doi: 10.1007/BF02859322

[B54] Zohary D. Hopf M. Weiss E. (2012). Domestication of plants in the Old World: The origin and spread of domesticated plants in Southwest Asia, Europe, and the Mediterranean Basin (New York: Oxford University Press).

