# Supplementary material for: Physical seed dormancy in pea is genetically separable from seed coat thickness and roughness
Source: Front Plant Sci. 2024 Feb 27;15:1359226. doi: 10.3389/fpls.2024.1359226 (PMC10927720; doi:10.3389/fpls.2024.1359226)
Supplement: Supplementary file 1 [file DataSheet_1.zip › Supplementary Figures 1-4 and Table 2.PDF]

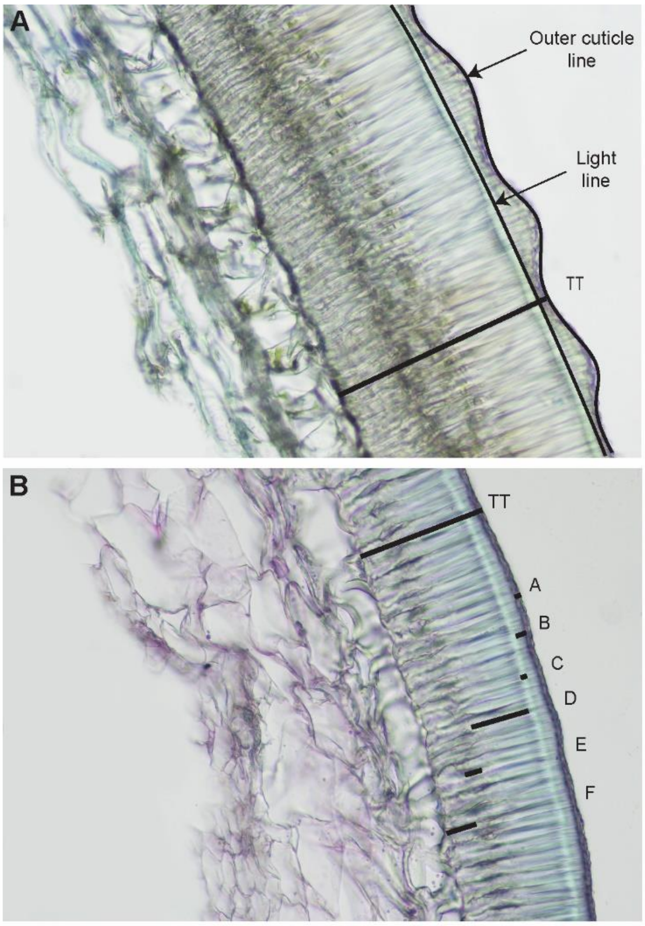

Supplementary Figure 1. Overview of testa measurements
(A) Representative photo of a pea testa with visible gritty bumps. Gritty was measured as the ratio of the length of the outermost line to the length of the light line layer. (B) Representative of a pea testa without gritty showing the different sections of testa measured from A to F – A (epidermins), B (macrosclereid layer from cuticle to light line), C (light line), D (largest section of macrosclereid cells between light line and dark deposit region), E (dark deposit region containing cell nuclei), F (macrosclereid cell section below dark deposit). Full testa thickness (TT) was measured as shown in both A (gritty) and B (non-gritty) lines. All measurements were done using ImageJ software (Schneider, C.A. *et al*. 2012).

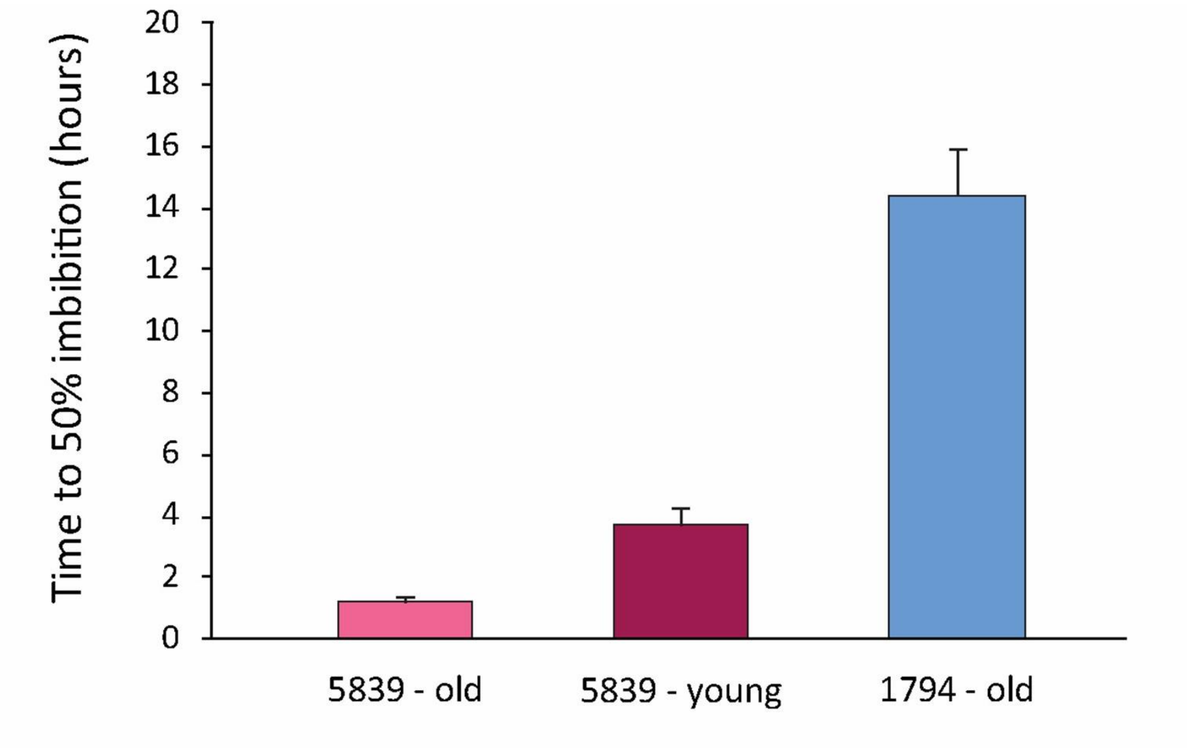

Supplementary Figure 2. Time to 50% imbibition.
Time to 50% imbibition in water for young (1 month old mature dried) and old (2 year old mature dried) seed from wild (JI1794) and domesticated (NGB5839) pea. Wild young seed had not imbibed within 100 days, after which point the experiment was concluded. This data was not included in the graph.

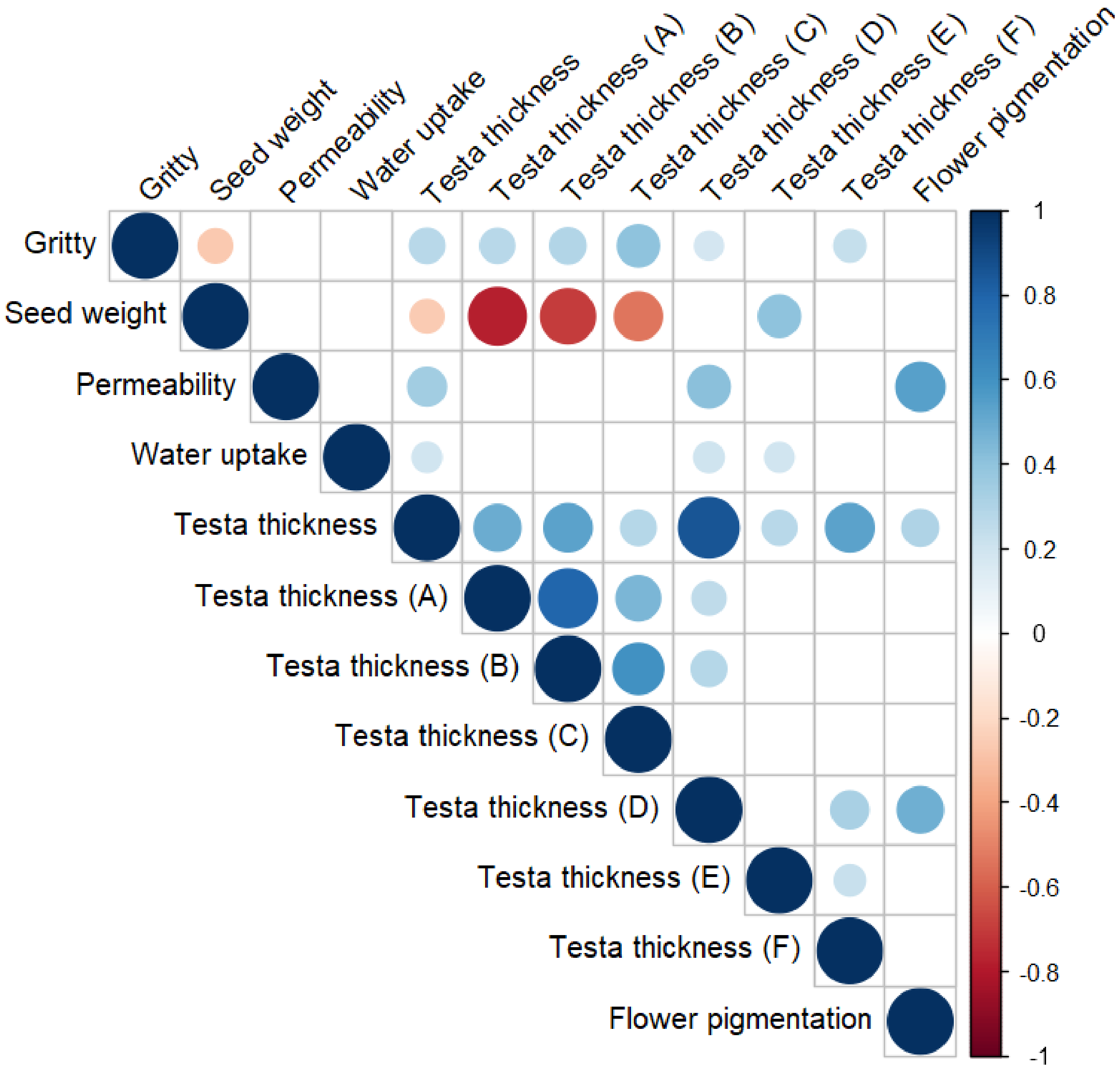

Supplementary Figure 3. Correlation matrix of all traits measured, using Spearman's Rank correlation coefficient. The colour and size of the circles indicate the strength and direction of the correlation. Only those correlations significant at p = 0.05 are shown.

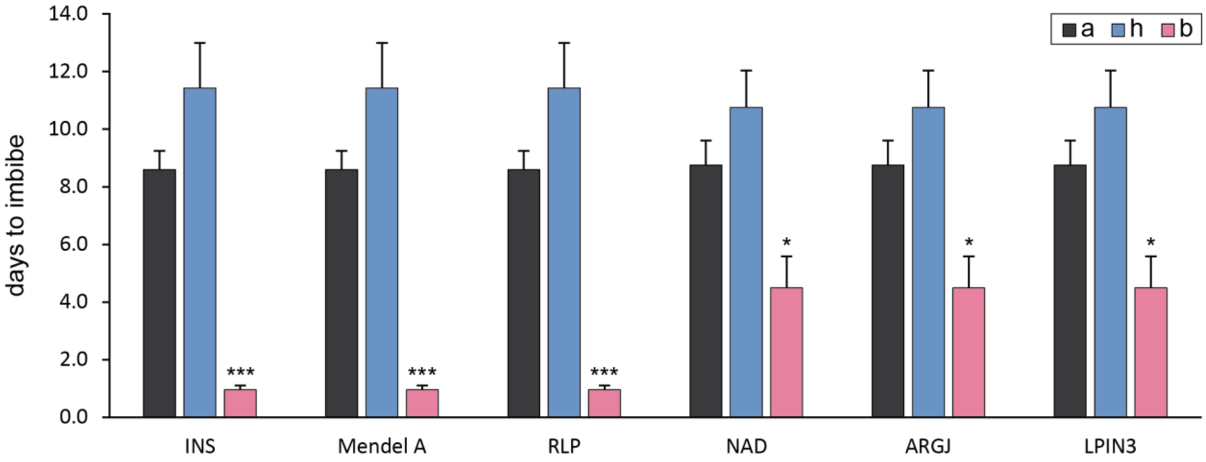

Supplementary Figure 4. The effect of segregating Mendel A region markers on permeability. Markers segregating across the region of Mendel A (See Fig.4 fine map). Markers to the left of INS were fixed (CWF and RPS27). Significant differences between a/h and b are indicated by an asterisk (*P<0.05, ***P<0.001).

Supplementary Table 2 - Details of gene-based markers spanning QTL peaks

			Primer sequence	
**Ps**	**Marker**	**Gene**	**Forward**	**Reverse**
1	RUG5	Psat1g073520	CAGGTTCGTTCTGAATCTTCG	CCTGCAGGAGTAAACGTGTG
1	MLO1	Psat1g099840	TGGCTCTTAGGCATGGATTT	TTGTGCATCATGTCCTGGAG
1	FTa3	Psat1g096760	TTGTTCTTGGAGCTGTAATTGG	CCTCAAATTTGGGTTACTAGGG
1	BFT	Psat0s3447g0040	GGCCAATTTTGCTGATGACT	TTTGACCACACTTGGTTCAAC
1	GA20ox	Psat1g113960	GACCAACTTTTTAAGAAAAGCA	TCTCCCATTTGAAAGAGCCTA
1	RNAhel	Psat0s3731g0160	GGGTTTGGTAGGTTTGGTAGAGG	GCATGTGCTATTTTTCTTCACTC
1	CABB	Psat1g111960	AGGATCTTCTTGCCTGATGG	CTTGCTTAGACCAAAAGGATCA
1	AGO1	Psat1g122920	TTACTCCCATGTCATCCTTGG	CAAGCATTAAAGAACCAGCAAG
1	FULa	Psat1g126560	AACCTAGTAGCTCTCACCGTAA	TTATATTATGGTGTTTGATTGATGA
1	FVE	Psat1g140960	GGAGACTCCTCCGTCGCAGC	TGTAGTTGCGCCTTTTTCG
6	INS	Psat6g059280	ACGAACTTCACTATTCCGACTTATC	GACCATTGGCTCCGAGTG
6	LPIN3	Psat6g069520	CGCCTACTTCTTTGGTCGAG	CCATGCATTGTAATCCTCCTG
6	RLP	Psat6g062960	GGTAACCCTAATTTGATTCATGTTC	GGCAATAACACAGTTGTATGCTTC
6	RPS27	Psat6g057640	AATCCACCTGTGGAACTCG	TTTGAGAGTGGCTAAACATGG
6	NAD	Psat6g066240	GGGGTTTGCTGAACACATTA	TGGGTGCACAAGAGGAATAA
6	CWF	Psat6g054520	TTGTCAGTATCCTAATAGTTTGA	CCAATCCATTGTTATGTCTCC
6	Mendel A	Psat6g060480	TCCAATCGAAGAACCTCTCG	GGGTTAGGAGTTAGGACAAACC
6	ARGJ	Psat6g067760	AAAAATCCGAGGGCAAGATA	CTGTGGATAGATGAGACTTGCAT
6	LF	Psat6g013960	GGTCCCTCTTTACCCTGGTATT	TGATCTGCAGGAAAACAATAAA
